# The weak link of democracy and the challenges of educating toward global citizenship

**DOI:** 10.1007/s11125-022-09607-8

**Published:** 2022-08-10

**Authors:** Piero Dominici

**Affiliations:** University of Perugia, Piazza Università, 1, 06123 Perugia, Italy

**Keywords:** Weak link of democracy, Simulation of participation, Illusion of a less asymmetric relation to power, Rules of engagement, Access to quality education, Democracy is complexity

## Abstract

Before discussing the prospects for educating young people toward becoming global citizens, we must ask ourselves: is global citizenship reality or illusion? What can be stated is that plain citizenship itself can no longer be considered merely a legal or judicial question. Today, citizenship is only partially linked to rights and duties deriving from the recognition of an individual as belonging to a community (local, national or international). Future citizens of the digitally hyper-connected global village face two dangers: simulation of participation and the illusion of having a less asymmetrical relationship to power. The rules of engagement are not being written by legislators but by agencies producing and sharing knowledge; citizenship (global or otherwise) is intimately correlated with access to quality education. Three concepts form the basis for educating toward global citizenship: awareness that citizenship and education are inseparable, awareness that democracy and education are inseparable, and awareness that democracy is complexity.

## Global citizenship: Reality or illusion?

Before discussing the prospects for educating young people toward becoming global citizens, we need to ask ourselves a difficult question: is global citizenship reality or illusion? In approaching this complex issue, what can certainly be stated is that plain citizenship itself can no longer be considered merely a legal or judicial matter. Today, citizenship is only partially linked to rights and duties deriving from the recognition of an individual as belonging to a community (local, national, or international). The global context, furthermore, is one in which the political systems of nation-states have become less and less relevant, with many modern democracies at risk of becoming “handmaidens” to the economic power system. Future citizens of the digitally hyper-connected global village are thus facing two tangible dangers: *simulation of participation* and the *illusion of having a less asymmetrical relationship to power* (Dominici, 1996, 2003). Today, the rules of engagement are not being written by legislators but by those agencies that produce, distribute, and share knowledge—once, but no longer, the nearly exclusive domain of educational institutions. In any case, the dimension of citizenship, global or otherwise, is intimately correlated to the access to and the quality of education and training.

This article will propose three fundamental axioms as the basis for considering the strategies for educating toward global citizenship:

1. Awareness that citizenship and education are inseparable.
2. Awareness that democracy and education are inseparable.
3. Awareness that democracy is complexity (see for example, Dominici, 1996, 2003).

Educational processes have a determining strategic relevance as the most indispensable instruments for rethinking and reconstructing a new global citizenship, a global citizenship constructed within a *culture of responsibility and transparency*, consisting of interdependent communities of people who have acquired awareness of the complexity that is our new habitat.

When we talk about educating toward a global citizenship, we must take care not to run the risk of creating a “citizenship without citizens” (Dominici, 2008). We would do well to avoid adopting the kind of short-term rationales that delegate education (along with everything else) to mere technology and digital connection. Connection will not suffice; what is needed are not merely connected citizens but citizens who have become capable of independent, critical thinking. Citizens who have not learned systemic thinking, who are not capable of thinking with their own heads, are the “weak link” of democracy. “Real” participatory citizenship—meaning active participation in cultural change—is always a complex production generated by bottom-up, grassroots social processes and mechanisms and cannot be taught through top-down, hetero-directed initiatives, even on a global level. If we want to encourage educators in all parts of the world to contribute to creating a concept of global citizenship, we must talk about a building a *new humanism* (Dominici, 1996). This new humanism, it must be clear, cannot simply retrace the footsteps of the previous historical and cultural experience known as humanism, which was based on the idea, vision, and perspective of humankind at the center of the world/universe. In fact, this concept is one of the original causes of the extremely critical situation in which we find ourselves today, regarding, in particular, environmental, ecological, and eco-systemic issues, as well as the very survival of our species and of our planet. New humanism must find a central place for humanity in a society that is, after all, made up of human beings, without forgetting that we are an integral part of the complex network we call *nature*. The arbitrary separation between culture and nature is just one of the many false dichotomies that are responsible for an artificial fracture between fields of knowledge, which will be discussed further on.

## Human beings: The non-measurable attributes of being human

Talking about new humanism means working on the social and cultural conditions for putting the “person” at the core of society. This cannot but lead to a series of questions related to our condition as human beings—or better, our condition of being human—and to the measurable (and above all, non-measurable) variables, indicators, and parameters that make this condition possible:

- What does the term “human being” mean today? (Dominici, 2014a, 2014b, 2019a, 2019b, 2019c, 2022; Popitz, 1995; Tegmark, 2017)
- What are the necessary preconditions for a new humanism?
- How can educators across the world contrast the current neoliberal educational mindset (which not only reinforces the misleading assumption that error is something that can be eliminated but also gives exclusive value to measurability, data, and statistics) with the *illusion of total control and predictability* (Dominici, 1996, 2003, 2017a, 2017b, 2017c, 2017d; Hammersley, 2013)?

Only by recovering the complex dimensions of educational complexity (Dominici, 1996, 2010, 2014a, 2016, 2017d, 2019b, 2019c) can we hope to build a new humanism and teach our children and ourselves how to inhabit complexity as true global citizens: this means teaching three fundamental aspects of EDUcation: *Error, Doubt, and Unpredictability.*

Naturally, it is necessary to view these strategic concepts in the framework of a more complex concept of society, calling for the reformulation of a “new social contract” (Dominici, 2003, 2008), which is often associated today with the term *global citizenship*. One word of warning, however: global citizenship can be all too “neat” a concept; it implicitly involves another “neat” concept that has been introduced in recent years: *digital citizenship*. To analyze these concepts critically, rather than adopting them as slogans in an attempt to render democracy less chaotic, less demanding, and less disorderly (while the very essence of democracy is to be found in its contradictory, conflictual, nonconforming sloppiness), we must first define *citizenship* itself, looking at its deeper meanings and implications. Can we speak about digital citizens, about global citizens, about inclusion and participation if we do not begin by educating the “person” to be first and foremost a citizen? And where can this take place, if not during the early years of school? In this phase of change, of a veritable *anthropological transformation* (Dominici, 1996, 2022), the crucial role of education must also include the accompaniment, mediation, preparation, and support of the mutations that have been sparked by the digital revolution and by the hypercomplexity of our civilization. An important part of this education should be directed toward the age-old issue of *teaching the teachers*, as well. This is undoubtedly part of what Edgar Morin called “thought reform”:

Thought reform would require a reform of teaching (in primary school, secondary school, university), that in turn would require a reform of thinking. Clearly, the democratization of the right to think would require a paradigm revolution which would allow complex thinking to reorganize knowledge and connect the fields of knowledge that today are confined within the disciplines. . . . Never before in the history of humanity have the responsibilities of thinking been so huge. (Morin & Kern, 1994, pp. 170–171)

Becoming a mature citizen means socially and culturally constructing oneself as a “person” to begin with, which is a complex process that must be activated at a very young age and be accompanied throughout the years by the joint efforts of family, school, and society, in order to reinforce what has become an extremely weakened social fabric, in which ethics and civil behavior (not only among the newer generations) have been cast off, returning only superficially as conditioned reflexes in response to media narratives and emotional triggers (*polarization* of positions). The so-called cultural question has its roots in these factors, which prevent us from actuating any kind of genuine innovation, that is, social and cultural innovation, with more open, inclusive social systems. This naturally has an important impact on the exercise of citizenship and participation. Only through well-designed and implemented educational strategies can we produce the level of cultural change that can set off economic, political, and social change; there is no room for improvisation or shortcuts. The strategic level for teaching begins during the earliest years of school: this is the crucial level where “well-made heads” are formed, and only here can a culture of legality, respect, and non-discrimination be forged, whereby the sociocultural conditions of a new humanism that will reduce the hegemony of the individualistic and egoistic value systems that have been weakening social bonds can be constructed.

Thus, the ethical issue concerning citizenship is threefold: it regards both education and freedom, which in turn requires responsibility (Dominici, 2003, 2008, 2010; Elias 1987; Jonas, 1979; Morin, Ciurana, & Motta, 2003; Nussbaum, 2010). The achievement of these dimensions will not be feasible, however, if students are not capable of critical analysis, systemic thinking, and using the scientific method, i.e., if they have not been taught how to use logic to develop or verify arguments, if they have not learned a method for synthesizing the enormous quantities of information they encounter, and if they have not received an education that enables them to see the connections between knowledge and life-experiences and to evaluate the social-historical origins of cultural norms and legal norms. Not only teachers and students but all social actors (i.e., people in general) must assume responsibility for their choices/decisions/actions – the actions they take and those they do not. Liberty entails responsibility (Dominici, 1996, 2003); if we do not become truly participatory citizens capable of “thinking with our own heads” and of working toward the social construction of change as active participants, rather than undergoing a top-down imposition, there will be no citizens in our global citizenry, only compliant, hetero-directed subjects. If that is the result that is being pursued, there is no need to insist on quality education or on teaching complexity and systemic thinking.

This is, of course, an extremely thorny issue—an issue that is all too often skirted or simply emptied through the use of media narratives, marketing techniques, or slogans but whose many implications need to be faced head-on. It can certainly be asserted that an extremely close relationship exists between the education we receive in schools and a truly active, participatory citizenry (Dominici, 2003, 2010, 2014a, 2014b, 2015, 2016, 2017a, 2017b, 2017c, 2017d), functioning in a less asymmetrical relationship with political and economic power. This is something each separate nation-state must strive for, even corporative social systems with limited vertical mobility and those based on clanship or crony systems, which are the most resistant to change, inclusion, and social innovation. It is not solely in so-called advanced societies that schools, education, and training have offered the greatest possibilities for social advancement or for improving the starting conditions of their young citizens; in more rigidly structured societies, this aspect is even more essential. When we mention schools, what we are talking about are our “social elevators”, which have, in recent years, broken down, depriving us of their vital functions. Alongside the widespread crisis in modern welfare systems, which is turning insecurity and precariousness into existential conditions for a large part of the world’s population, this has weakened the mechanisms of solidarity and has begun to undermine established beliefs in the rights of citizens, in people’s rights, and even in the right to knowledge.

Another crisis common to modern democracies, which would be automatically transferred onto a global democratic regime, is that in many societies, which are only apparently open and inclusive, opportunities are nominally guaranteed along purely theoretical lines, based exclusively on legal or judicial norms, that is, within a purely legal frame of reference. In other words, democracy is fast becoming mere procedure(s), a phenomenon that when transferred onto the kind of digital platform necessary for global governance, would represent even more clearly the loss of the human dimension, which is to say the person, the people, the system of relations, the educational and cultural contexts: in other words, the “life-worlds”. Further exasperated by the accelerations of the new digital velocity, which leave very little time for reflection, once again the danger is that of focusing merely on the technological and bureaucratic dimensions of social and political issues. Real inclusion is impossible under these conditions, and without genuine inclusion, any kind of innovation—be it technical or otherwise—will remain a “would-be” innovation.

Furthermore, the virtual space formed by the hyperconnected economic ecosystem, wherein money and information flow at breakneck speeds, has deprived politics and democratic regimes of the control of their own electoral bodies. The so-called process of disintermediation is separating political systems from their citizenry, thus from each single social actor, who becomes burdened with the impossible task of maintaining some kind of individual freedom, without the help of a community. Misuse of the concept of rationality is yet another factor that poses a tangible risk to the construction of a global form of democratic governance, along with the idea that there is no alternative to a self-regulating market economy, which has imposed a dominion-based rationale that permeates every aspect of social life. On top of this, the negative consequences of the exponential growth of financial power have become evident in the past few years. The crises of modern-day democracies, whose economic and technological spheres have all but overshadowed the social-political dimensions, are characterized by a gigantic loss of credibility and of trust in their institutions, owing to increasingly unequal social systems and evermore gaping divisions between the rich and poor, amplified by asymmetrical access to quality education and training. The correlations between education and inclusion, between education and citizenship, between education and democracy have never been clearer (Arendt, 1951, 1958; Banfield, 1958; Bellamy, 2008; Dewey, 1916; Freire, 1970; Marshall, 1950; Montessori, 1948; Norris, 2011; Piaget, 1970; Rawls, 1971). It should also be clear, at this point, that it is not, and never will be, technological and/or digital factors to determine citizenship or inclusion, or to create Montaigne’s famous well-made heads. The processes that have weakened the bonds that transform individual choice into collective projects and actions must be addressed by providing people with an education that teaches complexity and critical thinking (logic). There is an urgent need of informed and critically educated social actors in flesh and blood—active and aware recipients within their networks of social cooperation, who have reached full understanding of their rights and responsibilities as future global citizens, capable of taking solid, practical action to influence those making political decisions.

## Ethics and morals cannot be imposed

Taking for granted that the global dimension of civilization today is not something that can be undone, we are currently facing a social complexity that eludes the traditional systems of control and surveillance, and that requires a complete reformulation of thinking, working within a systemic perspective on the definitions and the constructions of a culture of citizenship and inclusion. No state can grow and be managed using solely economic paradigms; globalization itself has given us sufficient evidence of the fallacy of such an approach. The cultural question that has been eating away at our nation-states is even more relevant for a transnational entity, whose scope cannot be encompassed merely through a normative or legal framework but must be supported by collective and individual social actors who have grown up with the conviction that freedom and responsibility are related concepts. A shared ethics and a cultural model based on legality, civic-mindedness, and a full awareness of the rights and duties of citizenship are the building blocks and essential mechanisms for the very survival of any social or organizational system, not to mention one that aspires to form a global network. It is of paramount importance that decision makers in this ambient realize that *ethics and morals cannot be imposed* but must develop through real-life experiences and processes.

That a global citizenry runs even greater risks than separate nations when constructing a dimension of responsibility is one reason a different approach should be taken in combatting corruption and illegality, but above all in doing away with inequality and asymmetry. This can be confirmed by observing that even the most advanced modern democracies—despite being equipped with well-structured normative and legal systems (at times the complicated sets of laws, norms, and regulations are even too numerous to be effectively executed), professional codes, deontological guidelines, etc.—have serious problems of irresponsibility. This is a dimension that cannot be controlled by devices or charters, because it involves personal liberty and can only be understood from a relational angle. How can we globalize an assortment of societies in which individuals and corporations alike feel free not to answer to any kind of community for their actions? This question once again illustrates the crisis of educational institutions (not to mention the futility of (age-old) rationales based on repression and control), which have never attempted to solve the problems at their core, preferring short-term strategies, or better yet, emergency measures. What we are witnessing locally and globally is the legitimization of those who overstep rules, regulations, and shared social norms, along with a predominant avoidance of accountability that rewards those who place their specific economic interests before the common good, taking no notice of the precautionary principle. This attitude of rendering socially acceptable the infringement of laws and norms at times pervades even the very sites in charge of education and training. The inevitable (yet insufficient) result is to constantly fall back on stricter and stricter legislation, further limiting personal freedom, while at the same time failing to cope with the intrinsically complex nature of social systems. In all of this, following the triumph of the private high-tech giants, the “platform society” (i.e., the media, old and new, and in particular social networks, along with peer groups and marketing agencies) has literally devoured the spheres of communication, which in the past had at least been mediated by educational institutions.

## Coping with obsolescence: For an epistemology of error

If there is to be a global citizenry, it will consist of the inhabitants of a very complex global civilization; indeed, as has been said before, we will find ourselves inhabiting complexity itself. The future of this global complexity is, as all complex systems are, unpredictable. The predictions that are being made, paradoxically, reinforce this very unpredictability. According to recent statistics from the World Economic Forum, in the near future, 65% of today’s elementary school children will have jobs that not only do not yet exist but that we are not even capable of imagining at the moment. What kind of preparation or training can we propose for these children, what kind of skills or knowledge will they need, and how fast will these become as obsolete as the jobs we hold today?

The answer to this question is disarmingly simple yet profoundly significant. Young people should be encouraged to discover and follow their desires, their passions, and their imagination. Studies in both the humanities and the sciences (kept separate by the falsest of society’s false dichotomies) tell us that we must imagine, create, inspire, and be inspired; that we must observe, formulate, verify, or disprove through experimentation (and/or trial and error) a series of hypotheses that will form the basis of the kind of knowledge that will always be open to modification and even reversals. Regrettably, our educational systems have internalized a pseudo-scientific, zero-sum dogma that everything we learn, do, or learn to do must be useful in some way (Dominici 2018, 2020); must be measured, evaluated, and certified as something that will produce concrete results or provide economic returns; and must be predictable, controllable. A dogma which can be called *“the tyranny of concreteness”* (Dominici, 2008). Instead, schools should have the function of awakening and stimulating these passions; of guiding students on pathways capable of merging reason and imagination and of linking thought, action, and emotion—factors that are all but neglected in our current school and university curricula. The focus on facts and figures, on calculations and results, should be complemented by the teaching of a culture of error, an “epistemology of error” (Dominici, 2010), whereby the three aspects of education mentioned earlier (error, doubt, and unpredictability) should be encouraged and encompassed within the educational format, in the framework of a systemic view of ecosystems and of life itself (Arendt, 1958; Ashby, 1956; Barabási, 2002; Bateson, 1972; Bocchi & Ceruti, 1985; Canguilhem, 1966; Capra, 1975, 1996; De Toni & Comello, 2005; De Toni & De Zan, 2015; Dominici, 1996, 2003, 2010, 2014a, 2016, 2017b, 2017c, 2019a, 2019b, 2019c; Emery, 1969; Ferrarotti, 1997; Feyerabend, 1975; Foucault, 1988; Gallino, 1992; Gandolfi, 2008; Gell-Mann, 1994; Gleick, 1987; Holland, 1975; Israel, 2005; Kauffman, 1993; Kuhn, 1962; Maturana & Varela, 1980, 1985; Mead, 1934; Merton, 1965; Morin, 1973, 1977, 1980, 1986, 1990, 1991, 2001, 2004; Musgrave & Lakatos, 1970; Parisi, 1992, 2001; Popper, 1934; Prigogine, 1996; Prigogine &-Stengers, 1979; Simon, 1962; Taleb, 2012; von Bertalanffy, 1968; von Foerster, 1981; Wiener, 1948, 1950).

How can this be accomplished? To begin with, by ceasing to label, discredit, and stigmatize error, from elementary school onwards. Students learn at a very early age to avoid making mistakes and to fear taking any kind of risk or venture that might result in disapproval or low grades, whereas the primary objective should be learning by error and through error, rather than being taught that there is only one way to solve a problem, to tell a story, to form an opinion. Questioning “why” rather than “how”. What we are instead training and teaching the new generations to become is nothing more than efficient executors—executors of functions and rules who are incapable of reflection, incapable even of contemplating the nature of these functions and rules, incapable of asking themselves “why”. Both students and teachers have been trapped by these limited perceptions, resulting in an inadequacy that leaves them incapable of seeing the connections, the links, and the intersecting trajectories and loops characterizing the complexity we inhabit, whereas another primary function of education should be precisely that of teaching how to see and make connections. Ironically, considering that talk about complexity and systems thinking is all the rage today, it is amazing how much unawareness persists about the strategic significance of thought and thought systems, how blind we are to the ubiquity of complexity in every field and praxis, and how little we understand about the importance of error and about the crucial opportunity in being free to make errors.

## The grand illusions / complicated or complex systems?

What is more, the technological revolution—which has been creating and defining a new kind of rapport between individuals and the norm, between theory and praxis, and between citizens and government—has given citizens the illusion of sovereignty, of being masters of their own destiny, free to make their own choices, without realizing that, in reality, their digital involvement is merely a “simulation of participation” (Dominici, 2003) leading them to underestimate the importance of interactions, social interdependencies, and the physical communities they belong to. Media narratives on disintermediation have disseminated the myth of digital democracy, reinforcing an even more dangerous illusion, especially to potential global citizens: the illusion of having a “less asymmetrical relationship to power” (Dominici, 1996, 2003). But a hetero-directed individual is not a citizen; they are merely a subject. Thus, we run the risk, as mentioned before, of creating a *citizenship without citizens*. And certainly, regarding the concept of digital citizenship, it must once again be reiterated that no kind of digital citizenship is possible without guaranteeing the pre-requisites and the conditions of citizenship, without (at least) attempting to guarantee equality of starting conditions, whose absence renders all discourses about meritocracy purely rhetorical (Dominici, 2008).

Wishful thinking and illusions are not limited to individual citizens, however. On the part of institutions, technicians, managers, experts, and educators, there is a commonly held belief in an equally pernicious illusion: today many are convinced that total control will soon be possible, that through technology, algorithms, and artificial intelligence (AI), it will be possible to eliminate uncertainty and error from our lives and to predict future events and phenomena with mathematical accuracy. In short, there is a grand illusion of total control on the part of governments, institutions, and organizations. We know, however, (at any rate, we should know) that uncertainty is an integral feature of complexity, and that complexity itself is an intrinsic and integral characteristic of all living systems. Uncertainty implicates both error and unpredictability; uncertainty implies that total control is but a chimera. If for many this is still not clear, could it be that some confusion still reigns regarding the difference between complicated and complex systems?

Complicated systems are both manageable and predictable; it is possible to divide them into smaller parts for observation and analysis, and to put them back together to form a whole that will always be equal to their total sum. They are reducible and determinable (stimulus and response, cause and effect); thus, they can easily be reproduced. Their predictability guarantees the possibility of control. Complex systems, in contrast, can only be observed as totalities, focusing on the connections rather than the parts, which can never be reduced, controlled, or simplified. Complexity is innate to all living systems and collectivities, including groups of humans and their social systems (Coleman, 1990; Dominici, 2003, 2014a, 2014b, 2019a, 2019b, 2019c; Granovetter, 1973; Luhmann, 1984, 1990; Parsons, 1951; Putnam, 2000; Weber, 1922;). Recognizing and understanding complexity requires a systemic view of processes and dynamics, observing objects as systems rather than vice versa. In a complex system, the whole is always greater than the sum of the parts. And when observing a complex system, we need to remember that the very act of observing will affect the conditions, as will the parts (individuals and relations) themselves, which are constantly changing and cocreating the dynamics and interactions of and within their environment. It should be clear, however, that the opposite of complexity is not simplification but rather reductionism. The benefits and drawbacks of simplification will be considered later; suffice it to say that one thing that will never work is continuing to try to solve complex problems with simple solutions (Table 1).

**Table 1.**
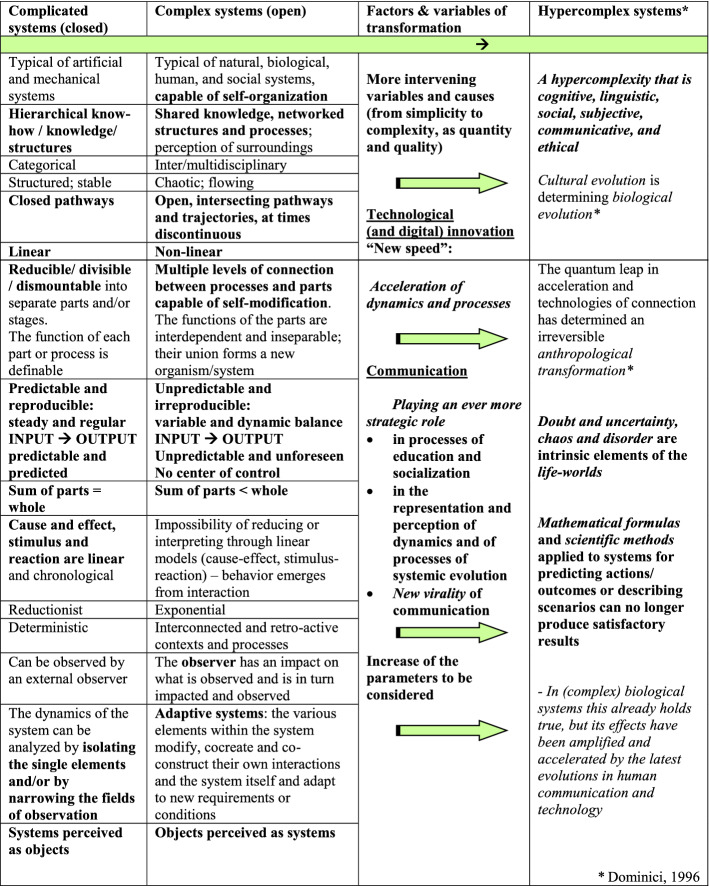
Complicated, complex, and hypercomplex systems

Up until this moment, therefore, technology has produced complicated systems functioning for and within living complex systems. In the near future, should AI make the kind of leap that is expected by its engineers and acolytes, redefining the interactions between complicated and complex systems, the scenario that in all likelihood will soon begin to play out is one in which AI is destined to become an epistemological and cognitive fracture, comparable to the rupture represented by the emergence of chaos theory in the recent past. If the prospect of AI interacting with living systems in a complex manner is imminent, no one can say what will come about, but it is so not farfetched to wager that it will itself begin to be much less predictable and controllable for its human creators. Which brings us to the next point: what does “being human” mean? And what is a human being, anyway? This is a question that had better be left to pure philosophers (certainly it is not something measurable or quantifiable). In any case, the possibility of creating machines that resemble human beings more and more closely is not excessively problematic, whereas the idea of human beings becoming more and more similar to machines is, quite frankly, an appalling prospect. Even in this era of hypercomplexity, brought on by an increase in variables, a change in parameters, an unprecedented acceleration of processes, and a virality of communication processes, which have literally *hurled us into a state of hypercomplexity* (Dominici, 1996, 2003).

## The great mistake, the overturn, and the need for hybrid figures

The biggest danger we are facing is due to the fact that we have given carte blanche to technology, in the mistaken belief that technology (in particular, the web) can solve any problem, including the capacity to bring politics and citizens back together. The “great mistake” (Dominici, 1996) being made by the hypertechnological civilization today, in fact, is precisely that of believing that education and culture (in particular, digital education and digital culture(s) can be solved by delegating everything to technical competence, speed, and simulation, to the “new” technologies of connection and the new ecosystems of communication, and hence that the kind of education and/or training that is needed today is purely technical and/or technological, solely a problem of “skills” and “know-how” and nothing more, which is the exact opposite of what we so desperately need. This kind of mentality will continue to reinforce the dramatic fracture that has separated studies in the humanities from studies in scientific fields, whereas the figures we need in order to educate for a global future are *hybrid figures* (Dominici, 1996, 2018, 2020). Hybrid figures who are aware of and open to the contamination among fields of knowledge and skills, hybrid figures who have completed educational itineraries based on interdisciplinarity and multidisciplinarity, designed to shape critical and elastic minds at every level, hybrid figures who are capable of recognizing complexity and connections, of evaluating the open architecture(s) of reality, and of perceiving limits and borders as opportunities for growth and experimentation.

**Figure 1:**
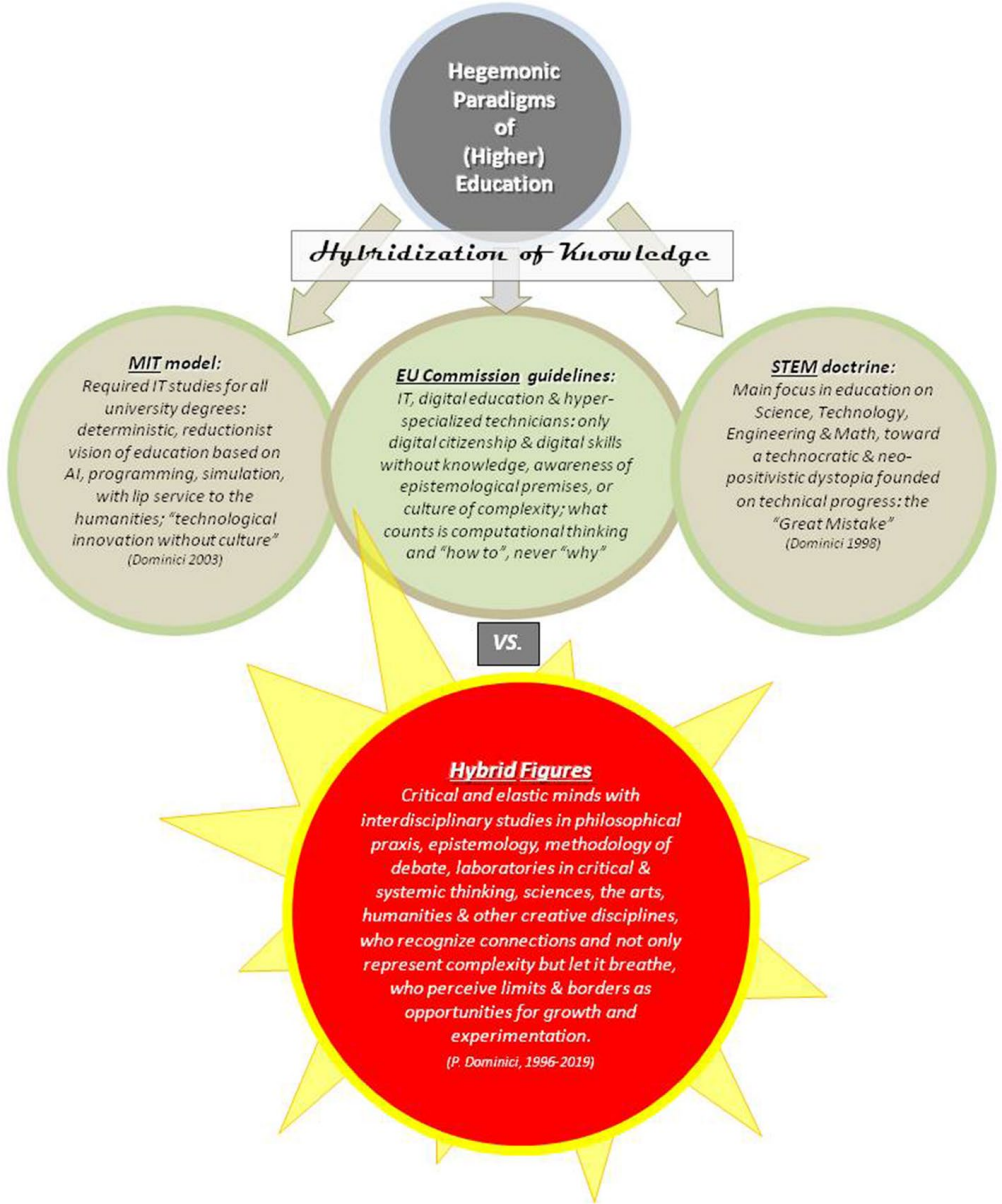
Hegemonic paradigms of (higher) education

Although today everyone is talking about contamination, hybridization, and elastic minds, at this point, it needs to be underlined that the concept of hybrid figures, developed by this author through years of study and research, is diametrically opposed to the hegemonic paradigms currently espoused in most spheres of higher education, supported in this delicate phase of transition by the media and the global system of communication, and generously funded by corporations and industries. These paradigms, which call for the “hybridization of knowledge” (as defined by enlightened minds at MIT and other great American universities, technological institutions, and centers) propose a required base in information technology for degrees in all disciplines as the fundamental basis for life and work in tomorrow’s world. It is difficult to deny that the MIT model is founded on a deterministic, reductionist vision of education (Figure 1). Based on AI, programming, automation, and simulation, with lip service to the humanities, it offers a “technological innovation without culture” (Dominici, 2003). It is similar to what is known as the STEM doctrine, which dictates that the main focus of education should be on science, technology, engineering, and mathematics (hence the acronym from the initials of these four words). This technocratic vision of society, based exclusively on science, technique, and progress, is part of the abovementioned “great mistake” and can lead nowhere else but to a neo-positivistic dystopia. Of course, it is the European Commission itself that recommends guidelines founded on information technology, digital education, and hyper-specialized technicians, where citizenship is only an issue of digital citizenship. Where only skills—above all digital skills—without knowledge, without awareness of epistemological premises, and completely lacking a “culture of complexity” are to be taught; what counts for the European Commission, apparently, is only computational thinking, in which the focus is on “how to” but never “why”).

These seem to be, at the least, very limited visions, deriving from a nearsighted, misleading, and deceptive perspective that, once again, takes for granted that what is needed for the hypercomplex society (Dominici, 2003) are solely technicians, programmers, hyper-specialized scientists, and as said before, mere executors. These universally acclaimed paradigms (the MIT model; the STEM doctrine; and the educational policies promoted by trans-political bodies, such as the European Commission) share a common postulate and numerous analogies, based on a belief in the omnipotence of technology and the benefits of progressively substituting excessive human contact with digital connections. In contrast, the hybrid figures this paper proposes stem from the idea/vision/perspective that the hybridization/contamination of knowledge and skills is an interdisciplinary, multidisciplinary, trans-disciplinary voyage of the mind and body through the humanities, the sciences (both social and “hard”), the arts, culture, music, and imagination. A voyage which must include the sharing of knowledge (rather than “knowledge sharing”), movement, rebellion, empathy, and contact with nature and with our emotions. Becoming a hybrid figure means completing disciplinary studies in epistemology and in philosophical praxis (not only in the history of philosophy, which is necessary but not sufficient) and in the methodology of debate; it means participating in laboratories on critical and systemic thinking; it means becoming able not only to represent complexity but to allow it to “breathe”. We must radically retouch how we think—that is, rethink thought itself, taking a systemic approach to education, which should be centered on forming minds built to reflect, to extract, to tinker, to grapple, to try out, to ponder, and to muse, based on observation, experience(s), experimentation, error, independent and critical thinking, logic, methodology, ethics, and epistemology.

**Figure 2:**
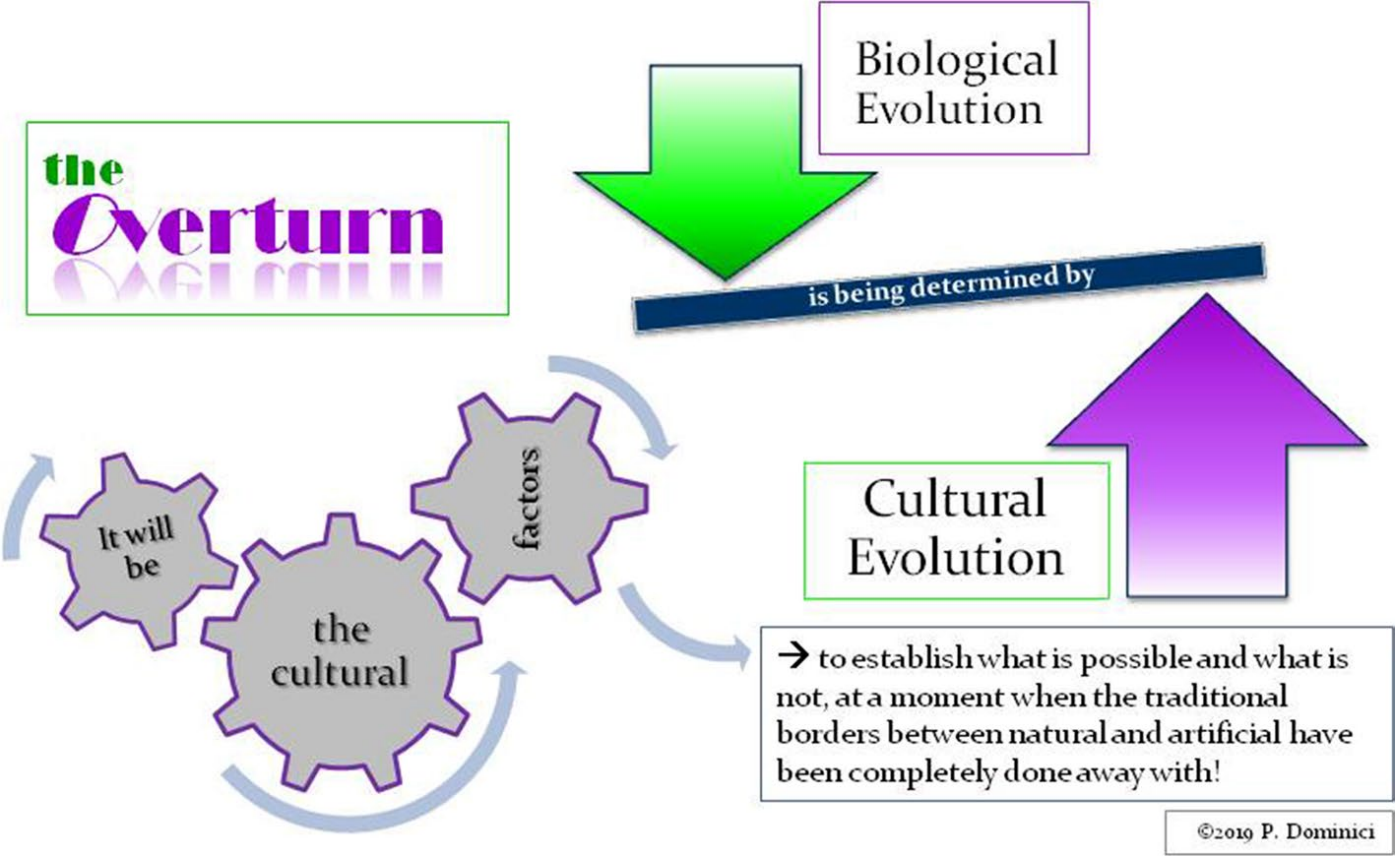
The overturn

**Figure 3:**
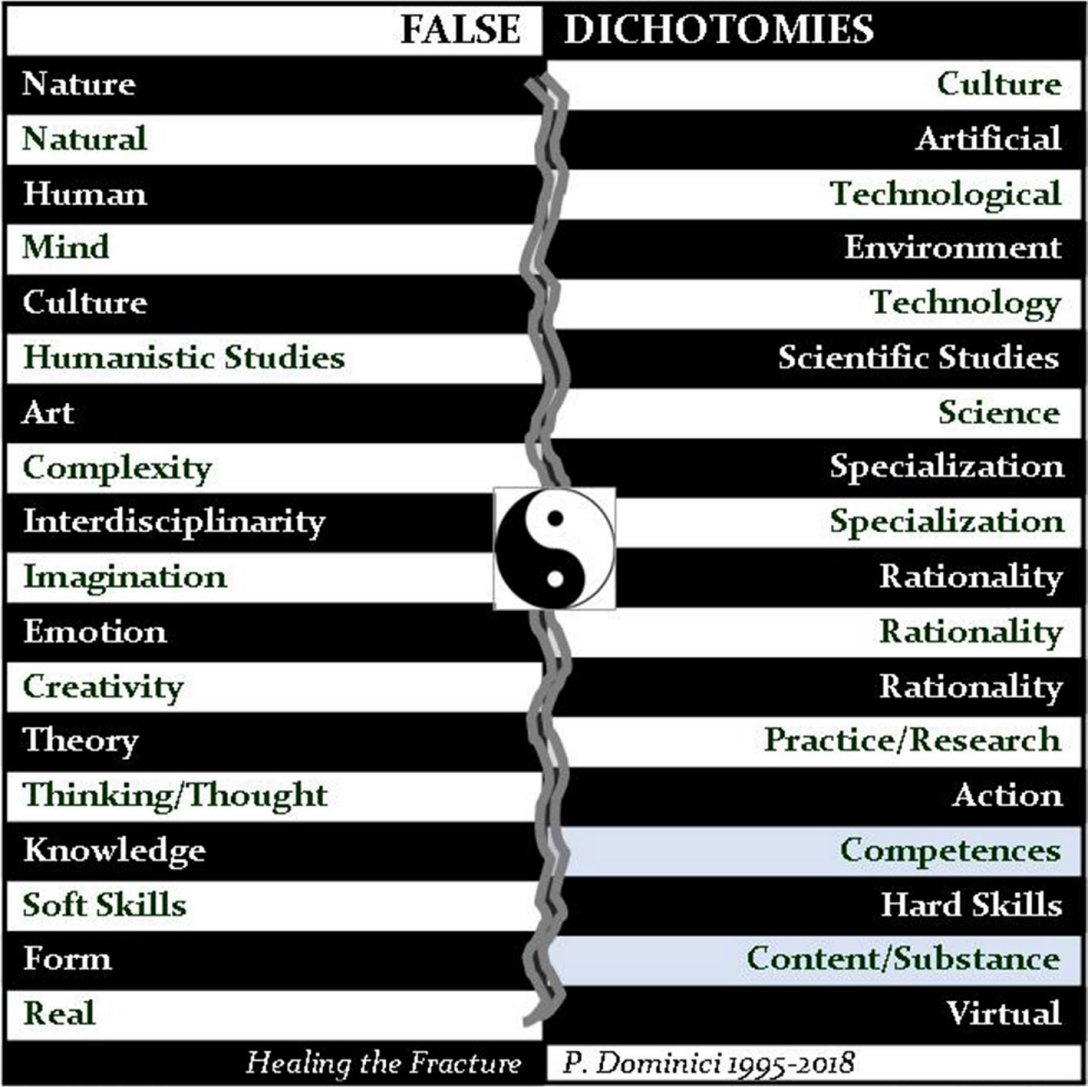
False dichotomies

**Figure 4:**
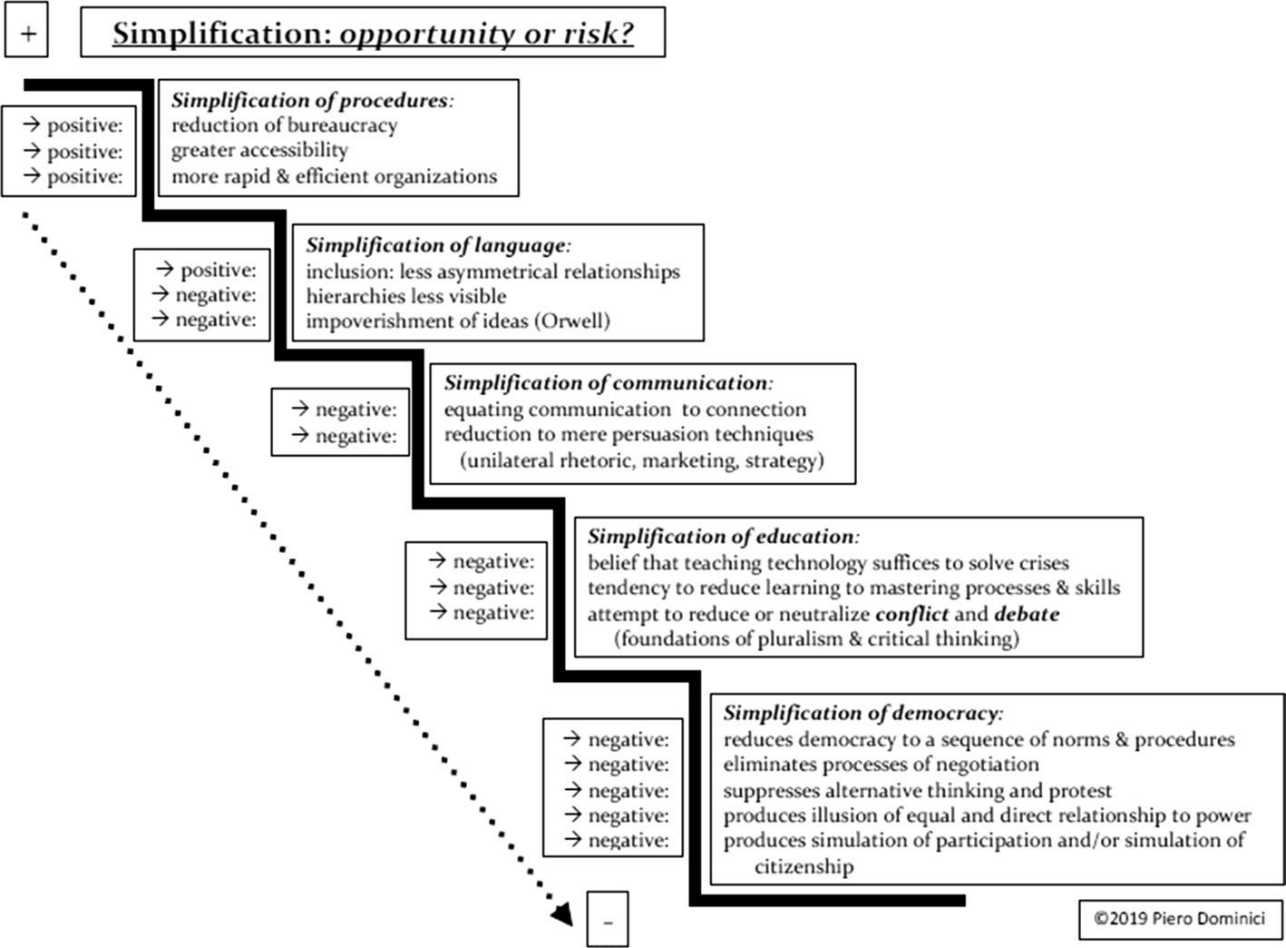
Simplification: Opportunity or risk?

Because technology cannot race ahead of culture as some appear to believe. Despite the accelerations it imposes, technology is always a product of culture and never something external to it (Dominici, 1996). Today, in fact, we are witnessing a complete reversal of the traditional evolutionary processes: we are in the midst of an “overturn” (Dominici, 2014a), where biological evolution is now being determined by cultural evolution rather than vice versa (Figure 2). From now on, it will be the *cultural factors* to establish “what is possible and what is not”, in a moment in which the traditional borders between natural and artificial have been completely done away with (Dominici, 2003; Morin, 1973, 2001; Prigogine, 1996) (Figures 3 and 4).

## False dichotomies

Along with the borders between natural and artificial, many other traditional borders have lost any relevance to the changes in our lifestyles, changes that amount to an *anthropological transformation*, brought about for the most part by the new speed of technological innovation and by many remarkable scientific discoveries, which have taken us to the brink of a quantum leap. The radical change in perspective that has become so urgent is being held back by the persistence of these many “false dichotomies” (Dominici, 1996), which continue to keep separate nature and culture, culture and technology, mind and environment, art and science, imagination and rationality, thought and action, form and content. If in the future there is to be a global civilization, that future belongs to those citizens, made up of people from different cultural backgrounds and aspirations, who will be capable of bringing together fields of knowledge, healing the fracture that has set technology against culture, humanities against science, emotion and creativity against rationality, theory against practice/research, complexity and interdisciplinarity against specialization, knowledge against competences, and hard skills against soft skills. The human and the technological, the virtual and the real, are all facets of the same entirety of existence, which, as physics tells us, cannot be distinguished or divided into waves and particles.

Therefore, among the preconditions of a new humanism is the recovery of the complex dimensions of educational complexity: “healing the fracture” between the human and the technological; rethinking the complex interaction/synthesis between natural and artificial; and rethinking educational processes from a necessarily systemic, socio-emotional perspective. Another is the construction of a “culture of error” (Dominici, 1996, 2010, 2016, 2017a, 2017c), teaching unpredictability and fostering the awareness that this is the fundamental element of our being human, not only of complex systems. Likewise, awareness of our incompleteness, of our limits and vulnerabilities—even in the framework of a renewed complex synthesis with technique/technology—to help support our fragilities and inadequacies and rediscover a humanism that has been purged of the ideology that saw the environment and ecosystems as mere instruments at our disposal is thus fundamental.

Far from being a consequence of the kind of healing unity being advocated, however, is the gradual drifting we are witnessing in our modern times toward a dismal flattening-out of controversy, an artificial reduction of debate and dissent, due to an almost pathological fear of any kind of conflict, which seems to be spreading further and further every day. But without conflict, criticism, disagreement, and debate, there can be no educational process; no achievement of critical thinking; and above all, no democracy, global or otherwise. Democracy, it should be obvious at this point, is complexity (Dominici, 1996). It can never be standardization, hetero-direction (Riesman, 1948), or conformism to a dominant point of view or narrative, however desirable or rational the latter may appear. It should be added here that another illusion that is wreaking damage on our society today is the ingenuous belief that more information, more connections, more and faster digital networks in this age of access / knowledge society (Benkler, 2006; Hess & Ostrom, 2007; Himanen, 2001; Rifkin, 2000) make our decisions and our procedures more rational. Yet all of this is nothing more than an example of limited rationality (Simon, 1947, 1959, 1962, 1997). Conflict and debate, when undertaken with respect for the opinions of others, are the very basis of that kind of education, based on the understanding of the value of error and doubt, that form creative and analytical minds, and should be carried out without forgetting that uncertainty is the underlying condition of human life, practicing an “epistemology of uncertainty” (Morin, 1973, 1980, 1986, 1991, 2001, 2004). We may strive to limit uncertainty as much as possible, but it will always be present, and at the end of the day, cohabitation with uncertainty is inevitable: “Uncertainty is the natural habitat of human life—although it is the hope of escaping uncertainty that is the engine of human pursuits.” (Bauman, 2008, p. 14 of introduction).

What is called for, in order to incorporate uncertainty and complexity into a new approach on the part of both citizenship and governance, based on “systems thinking”, are long-term policies in which plain citizens, educators, managers, and political decision makers can learn to cope with the unexpected, rather than recurring to ad hoc reactions and measures taken when emergencies loom. This kind of behavior amounts to a “culture of emergency” (Dominici, 2003), in which authorities and experts limit themselves to repeating slogans rather than actuating strategies- and to simply playing things by ear in an erratic and irrational manner, treating events as though they were simply occasional “black swans” rather than intrinsic aspects of the complexity of our ecosystem. Never has it become clearer how unprepared we are for coping with emergency; never have our inadequacies and superficial short-term politics been more obvious than in the face of a global emergency, such as the 2020 appearance of the Covid-19 pandemic, during which severely underfunded/defunded healthcare systems, media coverage based on fear rather than logic and reassurance, and an extremely punitive form of moral blame placed squarely and exclusively on the shoulders of the citizens brutally revealed the lack of systems thinking and coordination of our global civilization. The result has been a series of emergency measures, many of which were enacted without compensation or consideration in terms of economic suffering and cognitive/psychological distress, leading to a decline in solidarity, cooperation, and unselfishness on the part of citizens. And once again, digital technology has been proposed as the exclusive means by which all problems will be “virtually” solved, through a magical process of simplification.

## The opportunities (and risks) of simplification

Even in more serene times, simplification has been cited by many technicians, computational engineers, and digital gurus as an absolute value. It is quite possible, however, to challenge this view by considering this concept from a different perspective, which allows us to analyze whether what is termed *simplification* should be considered more of an opportunity or a risk.

Simplification is not, of course, an end in itself. As a means, however, its implications can be both beneficial and (more often) harmful. Simplifying procedures, for instance, is extremely positive. It helps to reduce bureaucracy; it provides more accessibility to a greater number of people; it streamlines processes and makes organizations more direct, rapid, and efficient.

As regards to language, the negative consequences of simplification appear to outweigh the positive. Admittedly, the simplification of language enhances inclusion, in particular by avoiding the kind of specialized vocabulary that tends to reinforce social asymmetry by dividing those who have received an education that has provided them with certain kinds of knowledge and competences from those who have not, thus making simulation more difficult to carry out in dialogue. But the use of simplified language can also be deceptive, helping to disguise hierarchies or making them more digestible, by giving the impression to less wary social actors that they are in on the game, and by permitting only a partial comprehension or analysis of the complexity of reality

Particular attention should be paid, in this regard, to the impact of linguistic simplification on the means and methods for consolidating global citizenship in the future. It can easily be seen that the use of English as an international language for communicating among different peoples of the world, while effectively breaking down barriers and supporting integration by providing a common framework for social construction, necessarily limits the rich linguistic diversity that is the direct expression of different cultures—thus eliminating variety, interpretation, and the capacity for fine nuances in form, manner, and expression, and indeed, all but eliminating entire linguistic continents from our cognitive geographies. This is a great loss to human cultural heritage and can rightly be considered one of the more insidious forms of colonization, a cultural colonization some would not hesitate to define a sort of *virtual subjugation*. Let us not forget, furthermore, the lesson learned from George Orwell in the last century: by far the most devastating consequence of simplifying language is the drastic impoverishment of ideas and of the very capacity for thinking them.

The pitfalls of linguistic simplification bring us to the closely related area of communication, whose best definition is “the social process of sharing knowledge” (Dominici, 1996). It is difficult to identify any positive effects of simplifying communication; what this generally means is reducing communication to marketing or persuasion strategies. Communication thus becomes mere rhetoric, losing its most essential and defining feature. At that point, we can be sure to have lost the other fundamental quality of genuine communication, the quality most neglected: the art of listening, a practice that appears to have all but died out in this age of perception management.

Furthermore*, communication* (complexity) is commonly confused with *connection*; currently, the two terms are taken to be substantially equivalent. Indeed, it would not be exaggeration to state that communication today is perceived as just an automatic function of connection, ensuring in this way that in organizations, institutions, and our hyperconnected global civilization, the prevailing vision is inevitably mechanized and mechanistic. As we know, both language and communication are intimately related to education, so it should come as no surprise that simplification is intrinsically damaging to educational processes. As mentioned before in speaking about the “great mistake”, our educational institutions have begun adapting to and adopting the prevailing concept of a need for simplification. In many cases, our schools and universities have lost sight of their raison d’être and are relegating learning to the mastering of processes and know-how (skills), perpetrating the belief that teaching technology, in particular digital technology, is a quick fix to our current educational crisis. We had better realize before it is too late that we cannot just keep chasing every technological transformation, just as we cannot remain oblivious to how deeply digital technology has changed our perception and understanding of reality, or ignore the ethical and epistemological implications.

As said above, even more dangerous for education is the attempt to limit or to neutralize conflict and debate, which are the very foundations of pluralism and critical thinking. If this pernicious form of simplification is allowed to prevail, and if fear of divergence succeeds in prohibiting us from engaging (civilly and respectfully) in conflict and debate, we will deprive ourselves of the possibility of doubt and diversity of opinion, which are the foundation stones upon which liberty and democracy are built.

Likewise, the implications of simplification on democracy are exclusively negative, if not downright catastrophic. Simplification of democracy can only produce a simulation of participation and a simulation of citizenship, hetero-directed (Dominici, 1996, 2003, 2008, 2014a, 2014b) from the top down. A simplified democratic system would fuel the illusion of having an equal and direct relationship to power. It would eliminate the processes of negotiation, suppressing alternative thinking and protest. The arbitrary concept that such a complex form of governance, delicately balanced between liberty and responsibility, can or should be simplified is based on an arid and soulless conviction that democracy is nothing more than a sequence of procedures and norms. This kind of obsession with simplification, standardization, surveillance, and control is based on the series of illusions mentioned previously. Democracy is complexity; it is a living connection of interacting and intersecting lives in continuous self-transformation. The future global leaders of the hyperconnected and interconnected civilization must be capable of coping with uncertainty and unpredictability, of processing and sharing knowledge, and of organizing it systematically and functionally. Access to resources (material and immaterial) must be guaranteed to all citizens in order to correct the asymmetries and inequalities of the new ecosystem in which we live.

The changes triggered by technological innovations, therefore, must be accompanied by social and cultural innovation, by an *inclusive innovation* (Dominici, 2017d, 2019b, 2019c). We should also take into account, however, that there is an intrinsic vulnerability in change, because in every sector and in every action as well as in every individual undertaking or collective praxis, innovating means destabilizing (Dominici, 2003), questioning consolidated fields of knowledge and methods Feyerabend, 1975; Weber, 1922), upsetting equilibriums, and breaking the chains of tradition (Dominici, 1996). Our job as educators is to help students learn to feel at ease with abandoning certainty and to move toward uncertainty, because the risks produced are also opportunities. Whether what we have in mind is educating the young to become global citizens or simply helping a small child discover the joys of reading, teachers should not shrink from helping to render systems, relationships, and the sphere of communication more vulnerable, at least for the moment. At the same time, education should be conceived of as a set of complex instruments capable of strengthening the effectiveness of rights and duties that are essential for the survival of modern democracies and of the concept of democracy itself, through the social and cultural construction of the “person” and the citizen, and thus of the public domain, which takes on a fundamentally important role, in consideration of the constant and rapid transformation of the global context of reference.
